# Supplementary material for: A genome engineering resource to uncover principles of cellular organization and tissue architecture by lipid signaling
Source: eLife. 2020 Dec 15;9:e55793. doi: 10.7554/eLife.55793 (PMC7771963; doi:10.7554/eLife.55793)
Supplement: Supplementary file 2. — The sequence of each oligonucleotide is provided. [file elife-55793-supp2.docx]

**Figure 1: Supplementary Figure 1: Oligonucleotides used in this study**

| **Oligos for generation of gRNA** |  |  |  |  |
| --- | --- | --- | --- | --- |
|  |  |  |  |  |
| chiRNA-anti | GAAGTATTGAGGAAAACATA |  |  |  |
| gRNA primer | G(N18)GTTTTAGAGCTAGAAATAGC |  |  |  |
|  |  |  |  |  |
| **pBFv-gRNA sequencing oligos** |  |  |  |  |
|  |  |  |  |  |
| seq-pBFv-vermF1 | CGGGGAATCAGAAAAGTGG |  |  |  |
| seq-pBFv-vermF2 | GACGCTCTCCTGAAACTTGG |  |  |  |
| seq-pBFv-vermF3 | CACGGGTCGCTTGTCCTC |  |  |  |
| seq-pBFv-vermR1 | CAATGAACTCAAACTGTTCTCCC |  |  |  |
| seq-pBFv-vermR2 | TACTGGAGCTAGTGCAGCGA |  |  |  |
| seq-pBFv-vermR3 | ACGGAAGTGGGCAAAATCTA |  |  |  |
|  |  |  |  |  |
| **Oligos for testing deletion** |  |  |  |  |
|  | Forward Primer-PCR1 | Reverse Primer-PCR1 | Forward primer-PCR2 | Reverse Primer- PCR2 |
| [CG2699](http://flybase.org/reports/FBgn0020622) | GGATTGGGACAATCAGATGAG | CACTAGGTAACGCGTGTTAC | AGCTTGAGGAACTGCAATCT | GAGTTAGAGAGAGTTAGGGC |
| [CG2929](http://flybase.org/reports/FBgn0037339) | AGATCGGGTAATCGGGGAAG | TCCGTGCTCCGTGATTACG | CCCGCTTATGCTCTATGTAT | GATGAGACTGTAGTAACCGG |
| [CG3682](http://flybase.org/reports/FBgn0034789) |  |  |  |  |
| [CG4141](http://flybase.org/reports/FBgn0015279) | CCAGATCTCTGATCATCTCG | CATGCGCCAATGTGCATGTG | GAGAGATTGGCGTGCTGATT | CGACACTATCAGCTATGGATC |
| [CG5373](http://flybase.org/reports/FBgn0015277) | GTTCAGCTTACGTCTTAGCC | GCAGCTCCATCATCTTGAAC | TGTCGCCGTTCGAACAGCTG | CCTGCATGTTTGCCCGATTA |
| [CG6355](http://flybase.org/reports/FBgn0028741) | AGTGGACTGATCTGGCGTTG | GTGTCCTCCTTCTCATGCTC | CTAACACCCCGACAACATGA | GACGCCTTGCTAATGAAACC |
| [CG7004](http://flybase.org/reports/FBgn0004373) | GCCTACAAGACTATCCGCAT | GTGTCTCGCGATCTCTCACA | GCCTACAAGACTATCCGCAT | GCTAGACAACATACACCTTACAC |
| [CG9985](http://flybase.org/reports/FBgn0016984) | GATAAGCAACAGGTGCTCC | CGTGTCTAGTTGGCAGTCAA | CCTACTCAAGCCCACTTACT | CGTGTCTAGTTGGCAGTCAA |
| [CG10260](http://flybase.org/reports/FBgn0267350) | CATTCGGTTAGGCGGCTCAT | CGACATCCACTTCCCTCTAA | CCACATTTGCTAGCCAATGC | GATTCGAGTACGGCGGTGTA |
| [CG11621](http://flybase.org/reports/FBgn0015278) | CCATCGAATGCTATCTCACC | CCGTGAATTCCCGATATGAC | GTTCTTCGTCCAGGTACTC | CATGTCGCGTCACTTCCAG |
| [CG17471](http://flybase.org/reports/FBgn0039924) | GGCAGAGAATTTCGATCAAC | CGGTGATCTTGGTACTACCT | GTCTTCATATCAGAGCACGTG | GTGTTGCACGAGCTAAGCAA |
|  |  |  |  |  |
| [CG3530](http://flybase.org/reports/FBgn0028497) | GCCTACCGTCAATGTTAGTC | CAAGTGTCTGTACATCCGGC | AGCACATCCCTAGTTCGTAC | CAAGTCTGCGCTCTGACATG |
| [CG3573](http://flybase.org/reports/FBgn0023508) | GCACTGAACACGATTCCACA | GTTGCAACTGAAACGGGGAT | CAGACGAAGACAAGTCACTGG | GTTGCAACTGAAACGGGGAT |
| [CG3632](http://flybase.org/reports/FBgn0030735) | CCGATTCACACAATGTCCGA | AGGAATGTTAGGACGCACC | AAGACTCCCAGCTGTTCGTC | GTGTAAACTCCATTTCGACCC |
| [CG5026](http://flybase.org/reports/FBgn0035945) | TGACGTACGCACTGCTGTG | AGTTGCATTGTGCTGTTTCG | CGTCGACAGAAGCAATCAAG | CCCAATCTGAGTCTGAGAGT |
| [CG5671](http://flybase.org/reports/FBgn0026379) | CAGAACGTACACTTTGCAATGG | GTCGAAGTTCGACAGTTCGG | GTCCAACGTGATACGCAATG | GAGTAGAAGGACAATGGCAG |
| [CG6562](http://flybase.org/reports/FBgn0034691) | GCATTGCAAACGACGAATCC | GCAATTGGGTTGAGTACGTC | CCACCAACAGATGTCTTAGC | CAGATGGAATCGGATCCTTG |
| [CG6707](http://flybase.org/reports/FBgn0036058) | GCGTCCGAAATTATGTGGCA | CTCTTCAACGGGGAGACCTT | GCAACTGGAAGTGCAGCCTA | TGTAGCTGAACTGCTGGAGA |
| [CG7956](http://flybase.org/reports/FBgn0038890) | GACCTCTGAAAGTCTCGAGA | GTGCAGCTTATTCTTGGTGTTC | CGGGCAATCGAGAAATCGTG | CTCACTACAGCTTGGTGCTA |
| [CG9115](http://flybase.org/reports/FBgn0025742) | CCGCCACTACATAATGTCCC | TCACAAGGAGCGTGCAACAG | TTGGCAGCACTGCTGCATGAG | TAGCTCCACTGAGCTGCGAA |
| [CG9128](http://flybase.org/reports/FBgn0283500) | CAACTGGTCCATCCCTATCAC | ACTCCACTTCTTCCCGATCG | GTGATAGCGACAGTGAATCC | GCTCGCCTAAGTGTAAGTGA |
| [CG9784](http://flybase.org/reports/FBgn0030761) | CGCTAGTCGATATCGTCATC | CTAGTGCCATGACACCGTAC | GCAACCCAGATTGGAGCAAC | GCGGGAATAGGTGGAATTGG |
| [CG10426](http://flybase.org/reports/FBgn0036273) | CAGTCAGCCCAATTAGCCTG | GCTTGTGCTTCAGAGTAGAGG | GGACCAAAGTCGCATTTGCA | GATCTACCGCAGCATTGCAG |
| [CG17840](http://flybase.org/reports/FBgn0031611) | CGAGATACACAAGTGACCAC | CAACCCACACCTGCTCAATC | CAACACTGGCCATTTCTCGC | GAACCTCTCCCAACAATAGC |
| [CG42271](http://flybase.org/reports/FBgn0259166) | CCGTCCATCTATGGAGTTGC | GTCGCGTGCAGGACTACTAG | GTCCGGCCACCAATTGTGTG | CGGTTGCCTCATCTGCAAAC |
|  |  |  |  |  |
| [CG3620](http://flybase.org/reports/FBgn0262738) | CATTTGCAGCTGCGTCGAGG | GGTCTAAACTTATCGGTGTACCG | CAGAAGTGCAGCAAGTGCTG | GATGTGGGCGAGTGTGTGTT |
| [CG4200](http://flybase.org/reports/FBgn0003416) | GCGGATGAGAACTGGATTCG | CTCCTGAATCAATCTCACGCAC | ACGTTTGGACCGGACAGGAT | CTTGATTTCAGTTCCAGGAGTTCAG |
| [CG4574](http://flybase.org/reports/FBgn0004611) | GTGGAACGCCTCTTTGACAG | GCTTGGATTCCCCTTAGCAG | GCTGTTGCCATACCACTAC | CTAATCGAGGTCAGTTCCCG |
|  |  |  |  |  |
| [CG5269](http://flybase.org/reports/FBgn0267975) | GAAGTAGCGCGAAAGGTATC | TTGCCGTACACTTGGTGTTC | GACTGTGGAAGAGGTAAGAG | GCTGCTGTGGCTTCACATTG |
| [CG11111](http://flybase.org/reports/FBgn0003218) | GACCAGGTCATAGACGACGT | CTTCAGGTCGCCTTGAGTTG | GCGGCAATCGATGATTGCCA | CTGGGGCCAGTTAAGAGATTG |
| [CG17818](http://flybase.org/reports/FBgn0027872) | TCTCCGGCATACAAATCCGC | CTAGGGAATATAGTTAACGCAAAC | GCTAATCAAGGAGTATCGCG | CCGTTCACTAGGATGTCTGT |
| PIP2 binding |  |  |  |  |
| [CG1063](http://flybase.org/reports/FBgn0010051) | CAACAGTAACGGTGCGTGTC | GTCCTCGAGGAGAGTTAAGG | CCTAGATGTAAGCGAACTCAC | AGCCCTAAGTTCTCTGAGTG |
| [CG2520](http://flybase.org/reports/FBgn0086372) | TGTGGTCAGCTCCTTTGGTG | CATAGGCTACCTTCCTTTAGC | GAGCGGCAGCAGAAACAAC | CTGTAGCATCGTGTAAACAGTT |
| [CG4032](http://flybase.org/reports/FBgn0000017) | GTAAGCGAATAAGCCGTAGC | CTCTGCACAGATCTGACTAG | CAACCACCCACTGGCAAATG | GCTACTACTACACGCTACCA |
| [CG4254](http://flybase.org/reports/FBgn0011726) | GTACATCAGTGAGGTACAGG | CTCTAGTGATCCAGCATGGC | CAAAGAGAGCACCCTGTATC | GGAAACGCGACTTGGTCAAG |
| [CG6948](http://flybase.org/reports/FBgn0024814) | GACCAAGTTTCCGGTTAGTC | GCATGACAACTGTGTGCACA | GTGCGTAGTGCCCAATTGTC | CGATGATCGCGGCTATGTTATAG |
| [CG7057](http://flybase.org/reports/FBgn0263351) | CGAGCTAAATCCCAAGAAGT | GTGTGCGGTATACGGCATAG | GGGATTTGAAGTGCCCAGAA | GACTTGCTGGAACTAATCTTGG |
| [CG7127](http://flybase.org/reports/FBgn0266667) | CGCGGTAATTGACAGTATGACC | AGCACCATCCACTAACTTCC | GCCAGTGTGCATAAATGCCTAG | GCGTGGCGTCTTCATTTCAC |
| [CG8532](http://flybase.org/reports/FBgn0028582) | CACCAACTGGTACCGACTCA | CTGACAGTGAAGGATTCCCC | GTAGCCCGAAACACACTTAAC | CTGGGTGTCGTTGCTGTTTC |
| [CG8604](http://flybase.org/reports/FBgn0027356) | CCTGCCCACACAAACAACTC | CATTCCTCATCTGTGTGTTCG | GGAGTTGCCTAAAGCGGCAA | TGGTTGGAAGCACTGCCCTA |
| [CG9012](http://flybase.org/reports/FBgn0000319) | CCGGTGACGCTGTCACTTTC | GGTAATTTTTGGCGCACATGC | GCATCAGTGCTGTCCATAAC | GCAAGTCCTACAAGTAGGGA |
| [CG9446](http://flybase.org/reports/FBgn0265935) | CAGAGCTAGACGTAGAGAAG | TCCAGGTCTGCTCGGTTATG | GCCACTGCAGCATCAAGATG | AACCCTATCTGTGCCACACC |
| [CG9579](http://flybase.org/reports/FBgn0000084) | GCAAACCGGAGAGCACTGAA | CTGACCTATGGCCATCATGG | GAGACCTCGGTGCAGTGC | GTCGGACATCTCGTGCTTG |
| [CG9968](http://flybase.org/reports/FBgn0030749) | GTCATTCTTGTGAGCTCTGTG | CCCTGGTCACTTTGTCAAG | GCGCCATGGAGTCAACTTGA | CTGTCGGTTCCCATGTTCAT |
| [CG10540](http://flybase.org/reports/FBgn0034577) | GCATCCGGTACAGGTACACA | CGACAATTGGTGGCGATCGT | CTTGGCCCATTAATTGCGGAG | CGGATGCTGTGGTGTTAATG |
| [CG10701](http://flybase.org/reports/FBgn0011661) | TGACCTCTATGGTCAGCAGT | GCAATAGGCTTTTGCCAGCG | GTGTTGTGTGTTCCCTTAGC | CTTTCTGGAGTCTCACCGCTC |
| [CG14296](http://flybase.org/reports/FBgn0038659) | CAGAACGTGCAGAGCAAGCG | CCGTAATCGTAGTAACCCGC | GGAGAAGGAACGGAGAAAAG | CTCTGGTGGCGAAACCAAC |
| [CG15015](http://flybase.org/reports/FBgn0035533) | GTCTGGAGCAGCTGCTCTGT | GTTAGGCGTGTTGTGTCTGT | GCACTGAGCTCGGTTCTATC | GGCTGTCCGACAGAACGCAAAG |
| [CG17158](http://flybase.org/reports/FBgn0011570) | CACCGAGAAAACCTCCAGAG | GTGGTGACAACAGTGCCTTG | TGGTAATCCCTGGTTTGCCG | CGAATATTGCTGCGCTCTTC |
| [CG18102](http://flybase.org/reports/FBgn0003392) | GAGGAGTGTGTGCAGTGAAG | ACTGGATGGGAATTGCCATC | ACGAGTTGGGATCGCAGTTC | GCTGGCTCTCTAAGTTTCTG |
| [CG31158](http://flybase.org/reports/FBgn0051158) | GTAGAAGAAGAAGCAGCGCA | CAGTGGTTCTGTGTGGATCT | GTTGGCCAAGCCCAAAACAG | CGTTCAATCGCCTGCTTCAC |
| [CG32434](http://flybase.org/reports/FBgn0026179) | GAAGTGAGATGGGTAGAGAG | GTACGCTGTGTGTGGGTTAG | GATCGTCGATCACCTTGTCG | CGTTCGGGTCGTTTCGATTC |
| [CG33653](http://flybase.org/reports/FBgn0053653) | CAAGTGACTGACGTACGTC | GGGACATATGTAGTTGTGCC | AACAAGTAATCGCGTTCCGC | ACCAACTTCCACTTCCTAAC |
| [CG44159](http://flybase.org/reports/FBgn0265042) | CAATCATCATCCCGAGCTCG | CATTCATCAGCTTAGCCTAGAC | GTGATCGCGTTGATCTTGCG | CAGATTGCCTCCACTTCAGC |
| PIP3 binding |  |  |  |  |
| [CG11628](http://flybase.org/reports/FBgn0086779) | GGTTACAAGATGCAGTGGCA | CAGGCATTGGTTCAACATGG | ACACAGAGCTCGCGTAACAC | GTAGAGAGCAACACAAGCGAG |
| [CG8743](http://flybase.org/reports/FBgn0262516) | CATGTTTCACCGTGCTGCAC | CTCGGTTCAAGTGCTTTGGC | GACTACCGATGAAAAGGCGC | ATGAAGACCTGCGGGTCGAC |
| degenerate binding |  |  |  |  |
| [CG2092](http://flybase.org/reports/FBgn0261385) | GACGCGCAGAATTTCGAATG | TCAGTGTACAATTGGCGTTAG | TATCGCCAACGTGGAGTGTC | GACGAACACGAACGTGTCCC |
| [CG11926](http://flybase.org/reports/FBgn0031640) | GTACTACCAAGGGAAAGCATG | CTCGAAAGTCCATCCCCGAAG | CGGACAGTGTTGGGAAATCC | AGCGGTGACTCGAACCTTTC |
| [CG10538](http://flybase.org/reports/FBgn0032821) | GAAAGGCCTGGAAACGGAATAG | CACGCTGCTCCAGAACTGTG | GTAGACTTGTAGACTTGTGGG | GAACATCGCTGTCACGTCGC |
| [CG8552](http://flybase.org/reports/FBgn0031990) | CTTCTTGACGAGCATACGTTTC | GGTCTCATGGGTGCCAATC | ACCGAAATCATTGGTCACCG | AGCTCAAGAACTTGCTACGG |
| [CG6760](http://flybase.org/reports/FBgn0013563) | GTTATGGCAGCGGTCACTGC | GAGCTAGCGCCTCCTTAACC | TGTCTCACCTGTTGCGCAAG | GAAGTCCACGCACTCATCAAG |
| [CG4114](http://flybase.org/reports/FBgn0004583) | GAGTACATGTTCGCAGATCC | CAGTGGCTCGTGTATTTTACC | GCTGAAAGATGAGACGTCCC | GGTTTCTCACGGTGGGTTTTC |
| [CG42250](http://flybase.org/reports/FBgn0261279) | CACTTGAACAGTGAGAGTTGG | GACAAGTTTAGCGATCGTGG | TGCAACACTGGACACGACAG | GTGAGAGTGGACATTTTGGG |
| [CG3779](http://flybase.org/reports/FBgn0002973) | ACACATTTTCCAGATCGCGC | GTGCTAAGCGCCATTGATGG | CGATTTCCGCTAACATCTGG | TGCGTGTGAGTAACTGTAG |
| [CG3715](http://flybase.org/reports/FBgn0015296) | CAGCTCCACCACTGAGCAAC | ACCAGGCAGGAAACTCGATC | CTATCGATATGCCTGCTAGC | GAACCCCAAGTCTGTGTTCC |
| [CG32677](http://flybase.org/reports/FBgn0052677) | GACAACACTGGCTGCTGGAC | AGGTACACACGCATATACATAC | GCAGTAATCACCATCACCAC | GGAACATGGACGTGGGCATC |
| [CG1513](http://flybase.org/reports/FBgn0033463) | TCAGGGAACGAGGCAGGTC | GATGACGTACCTTGTGCTCC | CTTTAGCCACGAGGACGCAG | GGCCAGATCGTATTCCTTGC |
| [CG9699](http://flybase.org/reports/FBgn0259923) | TGGCAACGTGCAGCAGTAAC | AGGCTGTGTGATGTCTGAAG | CTGCAACTTGCAACACAAGC | GGAGAGTCGTTGTCTTAATCAG |
| [CG44240](http://flybase.org/reports/FBgn0265194) | ACCACAGCGTGGTAGAGTAG | TGGCTGACTACATCTAGGAG | AGTTCCCAACACTTTGCCTC | GTGCATCGCATAACTCAGGC |
| [CG2774](http://flybase.org/reports/FBgn0031534) | AAGGTCCAGGATGGTAACGG | CTAACTAACTAGATCCATGGC | GCGTGATTACCGTTCCACATG | GAGAATACGCAGATCCCTGG |
| [CG6757](http://flybase.org/reports/FBgn0040475) | GAGAGTGCCTCTGTTCCAAG | CAGCGGGTTTGGTTGGATCATC | CAGGTGTGTTGGGGGAATAC | GCACTTGCACAAGTGGTTCTG |
| [CG8282](http://flybase.org/reports/FBgn0032005) | GCATTATGTCGCGTCTTGTG | CTTGATGTTGCCCAGTTACCG | AATTCCGTAGGAGTGAACC | CTGACTGCTCACAAACAGCC |
| [CG5658](http://flybase.org/reports/FBgn0004387) | CGCAACAGAGTGAATTGTG | GTCGCACATATGTTCGAGTG | AGTGGTCAGGCGAGAACAAC | CTGGTGGTCTTACATAGCTAC |
| [CG4006](http://flybase.org/reports/FBgn0010379) | CACCACAGGGAGCAACATCA | ATCACTTCAAGCGCTATGCC | CATCATCTACAGCCAATCCACC | CTGTTGCCCTTTCGTTCAACG |
| [CG1210](http://flybase.org/reports/FBgn0020386) | CAACGTATGAGTGTGAGCCAG | CTTACTTCTATTCCCGCCCTC | GTGCAAATCAAGGGAAGCAC | GTCTTCTTGCTAGGTGACGTC |
| [CG14991](http://flybase.org/reports/FBgn0035498) | ACGGTGCTGAACGTAAGTTG | GTGTGGTCCACAGATTACC | ACGTCATCGTTTCGAGGGCA | TCAACGCACCCTACATTAGG |
| [CG7729](http://flybase.org/reports/FBgn0036688) | CGATGCAATTGTCCGGATCC | GGTCTTTGATCCACTTGGTAC | GGCATAGTCAACGCAATTGC | GCTAAGGCCACGGTTAAGTC |
| [CG8176](http://flybase.org/reports/FBgn0037702) | GACTGTTGCACCATCCTGAC | GGTCCCACTTTCGATTTCGT | GGATGAGAGACTTAATGCGGA | TGTCGTGCTTCGATCTGTGG |
| [CG14228](http://flybase.org/reports/FBgn0086384) | AATGAGGCAGTCCGCGATAG | GAACTGCATGGAAGGTGTTG | GAATCGTGCCACTAGATGGC | ACGGTATCTAATCGCCCATC |
| PI3P binding |  |  |  |  |
| [CG14001](http://flybase.org/reports/FBgn0043362) | GTACTCCTAGCCAATAGCAG | GACCACTTTTGTAGTCTGGTC | GATCACATGAGGAGTACGCTC | GGTCGGTCATATATGCTTCG |
| [CG4030](http://flybase.org/reports/FBgn0034585) | GGTATGAATCACAAGACCG | CAGTTAACTGAGCCATCCAAC | CAAGCAGATAAACCTGGTTGG | CATGGACTCGTGTAGTTGTC |
| [CG15667](http://flybase.org/reports/FBgn0026369) | AAGATAGAACGAGATAGCATC | CTGAGCCATCCAACAATACC | GTTATGACTATATTCGAGGTGTG | CATGGACTCGTGTAGTTGTC |
| [CG2903](http://flybase.org/reports/FBgn0031450) | TTCGTCATCTGGCAGCACTG | AGTGGCTGCTCTTTCGGATG | GCAGCACTAGCGCATTGCAG | TCGATCTCGTGCAGCACTAC |
| [CG11807](http://flybase.org/reports/FBgn0033996) | CAAATGTCCATCGACCTCTG | AAGTGAAGTTGACTGGCCCG | ACTGCTGGCAATACTGGCTG | TATCTCGGTACCACTTACTATG |
| [CG3860](http://flybase.org/reports/FBgn0034951) | CTTCCACACCCAAGATGGCA | GATTGACTGAGGTCAACAGC | GACTACGCAGAGCATGTGGA | GATCGTACAATCACGTGGTC |
| [CG14782](http://flybase.org/reports/FBgn0025381) | TTCGACTCGGATTCGGACTC | ACAAGGTATCGGGATATGCG | GATCGAGCTAGCTGAGCAG | CATTCGCTATGTGGTCCGTTC |
| [CG41099](http://flybase.org/reports/FBgn0039955) | CTATCGCAGATTTCCGTACG | GACCGGTTTTCAAGTGTCAC | CGCTACATTGCAGTATTTCGGG | CTATGACAAGGAGCCATTTCC |
| [CG8506](http://flybase.org/reports/FBgn0261064) | ACTCTGACCGTATTAATCGAG | CGTAACGTAAGCCATGTAATTTGTC | AGGACTACAAGTGCCGTAAG | GTCATAGAGGGAATGAATATAC |
| [CG6410](http://flybase.org/reports/FBgn0034265) | CACACTGCGCTCCACAAAAG | CAACCCCTCGATATGCACTTG | GTCATGTAGCAAACGAAACGC | GTTGCGTAGAGTTATGCTCAG |
| [CG5734](http://flybase.org/reports/FBgn0032191) | CACATCTGGGCGAAAAGTGAAC | CGTGCCATTCATCTTGAGC | GACAATGCAGGTGTGATCCG | GCTGCGCAAATCCCACTTAG |
| [CG32758](http://flybase.org/reports/FBgn0052758) | CAACGCTACTGCGAGATGTC | TGCAGCACCTTCCGCCTTC | GCATCCATTATCGACGCTATC | AAGCAGTCCGCAAGGAATGC |
| [CG6359](http://flybase.org/reports/FBgn0038065) | GAATAGTACCAGAATCCGGG | GAGGGGCTCAACATTAACTG | GTCTATAGCTCTGCCGATCC | GCAAGCACACACATTCACAC |
| [CG6116](http://flybase.org/reports/FBgn0032499) | CAGTGTTTTGCAGCACTTATGC | GATGGCTAACATCTGCACC | ACAATGATCTGGGCTACGGG | CGTGACTCGCTCGTGTATTG |
| [CG5168](http://flybase.org/reports/FBgn0032246) | GCAGCATTCCTAAACTTGGCG | AATCGATGGATTCGTGTATTTATG | CTAGACGAGCTTTCCAACAAC | GTTAAGACCGAAACGCCTCGTG |
| [CG7986](http://flybase.org/reports/FBgn0035850) | GCAGTCCGATATAGCTAACG | ACGGGATCGTCACCCTTCAC | AACCATGATGAGCCTGCTCG | CTGCACTTGCACTGCTC |
| [CG34399](http://flybase.org/reports/FBgn0085428) | GTGTTGCTCCTGTGTAATCG | GGGACACACACTCAACAATG | GCCCACTTCATTGGCATGTG | CCCGTTGCTTATGCAAATGAG |
| PI4P binding |  |  |  |  |
| [CG9113](http://flybase.org/reports/FBgn0030089) | GATAGCTAGACCTCGAGCC | CGATTGCGTGGGTGTCTCTG | CCTATGTGAAGCACTCGCCG | TCCGATATTAGGCTAATCTGG |
| [CG7207](http://flybase.org/reports/FBgn0027569) | TGAGTCAGGCGATGCGATC | ATGTAGCTATGCATCCCTCAC | CACGATAGGAGCTCCCATC | TGAGGCCACAAACTGGAGGC |
| [CG3002](http://flybase.org/reports/FBgn0030141) | CATGCAATGGTGGTAGAAATG | GGGGAGCACAACATAGATGC | GCTCGCCAAACTATCGATAG | GTCGGGGTATTTTAGTCCAC |
| [CG7085](http://flybase.org/reports/FBgn0267378) | GGCCAAACCATAGACTTGCT | CTCTATGTGTCGTGGAACAC | GATGCCCATCCAGGCCAATC | GGACAATGGAGCGTTTGATTGG |
| [CG2078](http://flybase.org/reports/FBgn0033402) | TCACTCGGTCCATTTCGCAC | GATACATTTGTGCGGATATGTGG | ACAGAGCAGCACAACTCACG | GTGACGACATTGGTGACTGG |
| [CG6708](http://flybase.org/reports/FBgn0020626) | TCGGAATCTCGGTGGTGATC | GTGTGGAGGCTGGATATCTG | GCCGTCCAATAACCAATAGC | CTGCTATGTGTGGTAGTGGC |
| PI5P binding |  |  |  |  |
| [CG13398](http://flybase.org/reports/FBgn0032042) | CAAGTTAGCATGCCCGTTGC | GCTATCCATTTGATCTGCTC | CACAATTGCGTTCTGTCGGG | CTAACAAATCTTGTGGCCACTG |
